# Circulation patterns associated with trends in summer temperature variability patterns in North America

**DOI:** 10.1038/s41598-023-39497-5

**Published:** 2023-08-02

**Authors:** Chibuike Chiedozie Ibebuchi, Cameron C. Lee

**Affiliations:** 1 Department of Geography, Kent State University, Kent, OH USA; 2 ClimRISE Lab, Kent State University, Kent, OH USA

**Keywords:** Climate sciences, Environmental sciences

## Abstract

This study improves the understanding of circulation patterns associated with regional temperature trends by characterizing boreal summer temperature variability patterns in North America using rotated S-mode principal component analysis. We analyzed gridded observational 2-m temperature datasets and the ERA5 reanalysis temperature dataset to examine the climate patterns associated with long-term trends and inter-annual variability of temperature variability patterns in North America. Our analysis revealed significant trends among some classified temperature variability patterns from 1979 to 2022 summers, with inter-annual amplitudes (i.e., a departure from the mean state) signaling toward the warm regime. The anticyclonic circulation anomaly over the temperature coherent regions associated with Greenland/northeastern Canada, and Alaska, respectively, is linked to an increase in warm air advection and above-average temperatures, while cyclonic circulation over the northeast Pacific coast enhanced warm air advection and temperature increases in the coherent region comprising the northwestern portion of North America. The increase in global mean land and ocean temperatures is strongly associated with the long-term increase in the amplitude of atmospheric circulations associated with warm regimes in parts of North America. At the interannual time scale, temperature increase over Greenland/northeastern Canada is strongly associated with the negative phase of the Arctic Oscillation. These findings highlight the modulating effects of global temperature increase and warming of the western tropical Pacific Ocean on the increasing amplitude of circulations associated with warm regimes in North America. Our results further indicate that the enhancement of anticyclonic circulations over the Arctic contributes to nearly 68% of the observed reduction in sea ice extent.

## Introduction

Climate trends are a fundamental aspect of climate research, as they serve as valuable indicators of climate change^1,2^. However, climate change can also manifest as abrupt changes in the mean state of the climate, which may persist for several decades^3^. Climatic trends, such as the observed increase in the Earth’s temperature and warming of the upper ocean, have been shown to be largely caused by anthropogenic forcing^4,5^. This human influence on the climate system can alter the frequency and/or strength of weather patterns, leading to changes in day-to-day weather variability^6^. On the other hand, internal forcing such as anomalies in large-scale circulation modes that oscillate between high and low index values can cause abrupt shifts in the mean state of the climate system^5^, and when such anomalies synchronize with the direction of external forcing, accelerated climatic trends become plausible^7^. Identifying the underlying climate modes of variability responsible for long- and short-term variability in climate variables is essential for improving the accuracy of climate predictions^8^. Understanding these climate modes can provide valuable insights into the mechanisms driving climate variability and change and can help inform effective strategies for climate adaptation and mitigation. Given the severe impact of temperature extremes on human health^9,10^, the economy^11^, and the ecosystem^12^, it is crucial to understand the underlying climate modes associated with long-term changes in temperature variability patterns in North America. This region is frequently impacted by heat waves and cold outbreaks^13^, making it an important area to study. In this study, we will analyze these climate modes of variability, focusing on temporal changes in atmospheric circulation variability.

Several studies have addressed temperature trends in (parts of) North America^10,14–17^, and the possible circulation patterns (or weather types) driving the trends^10,18^. A review by^19^ highlighted that temperature extremes in North America are commonly linked to large-scale displacements of air masses. Moreover, studies have highlighted the linkages between teleconnection patterns and seasonal temperature variability over North America^20–22^. Reference^21^ reported a strong correlation between the Pacific North American Pattern and winter temperature over North America. Reference^23^ highlighted that the North Atlantic Oscillation (NAO), impacts synoptic weather-type frequencies in parts of North America. Reference^24^ found widespread significant correlations between the Western Pacific (WP) pattern, the NAO, and cool/warm weather types in parts of North America. From December to March^25^ reported that the El Niño Southern Oscillation (ENSO) correlates with above-average temperatures over Alaska and parts of Canada. Other studies reported on other teleconnections modulating regional atmospheric circulation patterns over North America and beyond^26–29^. For example, the tropical Pacific SST shows the strongest impact in winter as that's when ENSO generally can reach its maximum strength. The anomalous heat flux causes the jet stream to shift from its' climatological position. The extratropical cyclones also exhibit variability during El Niño and La Niña, which contributes to temperature and precipitation variability in the mid-latitudes^26–28^.

Temporal changes in atmospheric circulations exhibit spatial heterogeneity, largely because different regions around the globe respond differently to external and internal climate forcing. This variation can be due to a combination of factors, including differences in land surface characteristics, atmospheric circulation patterns, ocean currents, and regional climate feedback^30^. In the case of North America, no prior study has comprehensively addressed the climate patterns associated with temporal changes in atmospheric circulation variability of regional circulation patterns associated with warm/cold regimes over relatively temperature-coherent regions. Thus, the major contributions of this study are: (i) characterizing temperature over North America into distinct variability patterns or relatively temperature coherent regions; (ii) examining the circulation patterns linked with warm and cold regimes over these regions; (iii) exploring trends in temporal changes in atmospheric circulation variability; and (iv) identifying the climate indices associated with long- and short-term variability in the atmospheric circulations. This study aims to provide a better understanding of the complex interactions between temperature, atmospheric and oceanic teleconnections, and climate change in North America. The improved understanding of circulations patterns associated with temperature variability in North America facilitates the development of climate model simulations toward a more accurate representation of the climate system and more reliable future projections of climate change in North America.

## Results

### Temperature variability patterns in North America during JJA

**Figure 1:**
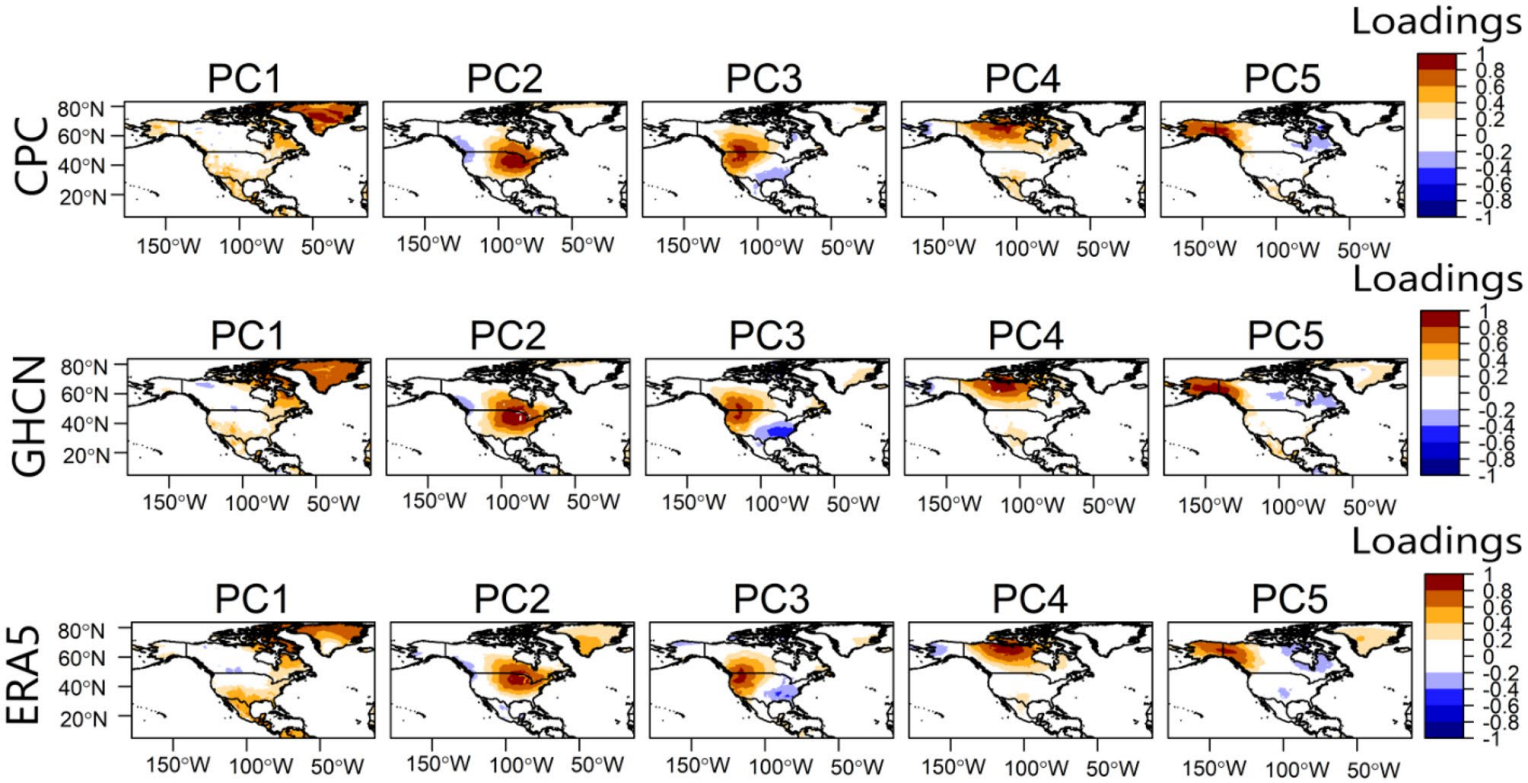
Temperature variability patterns in North America during JJA, from 1979 to 2022. The first row is the first five PCs from CPC 2-m temperature; the second row is the first five PCs from GHCN 2-m temperature; and the third row is the first five PCs from ERA5 2-m temperature.

The rotated S-mode PCA indicated that 5 physically interpretable temperature variability patterns can be classified in North America during JJA. The temperature variability patterns are shown in Fig. 1. The optimal number of variability patterns considered physically interpretable was decided by matching the rotated PCs to the correlation vectors to which they are indexed and requiring that all retained PCs have an adequate congruence match (that is, greater than or equal to 0.92). The varimax rotation algorithm rotated the PCs to fit the correlation patterns, and the results of the fit and the number of PCs to retain were consistent between the two observational datasets (Table 1). The explained variance by the PCs is 30.13% for PC1, 16.26% for PC2, 13.81% for PC3, 7.88% for PC4, and 7.48% for PC5. Thus, the five retained PCs explained a total of about 75.56% of the 2-m temperature data.

**Table 1:** Congruence match between the retained PCs, from the JJA temperature data, and the correlation vectors that have the largest loading magnitude on the PC in question.

PC	CPC	GHCN
PC1	0.96	0.96
PC2	0.98	0.98
PC3	0.96	0.92
PC4	0.92	0.95
PC5	0.95	0.96

**Figure 2:**
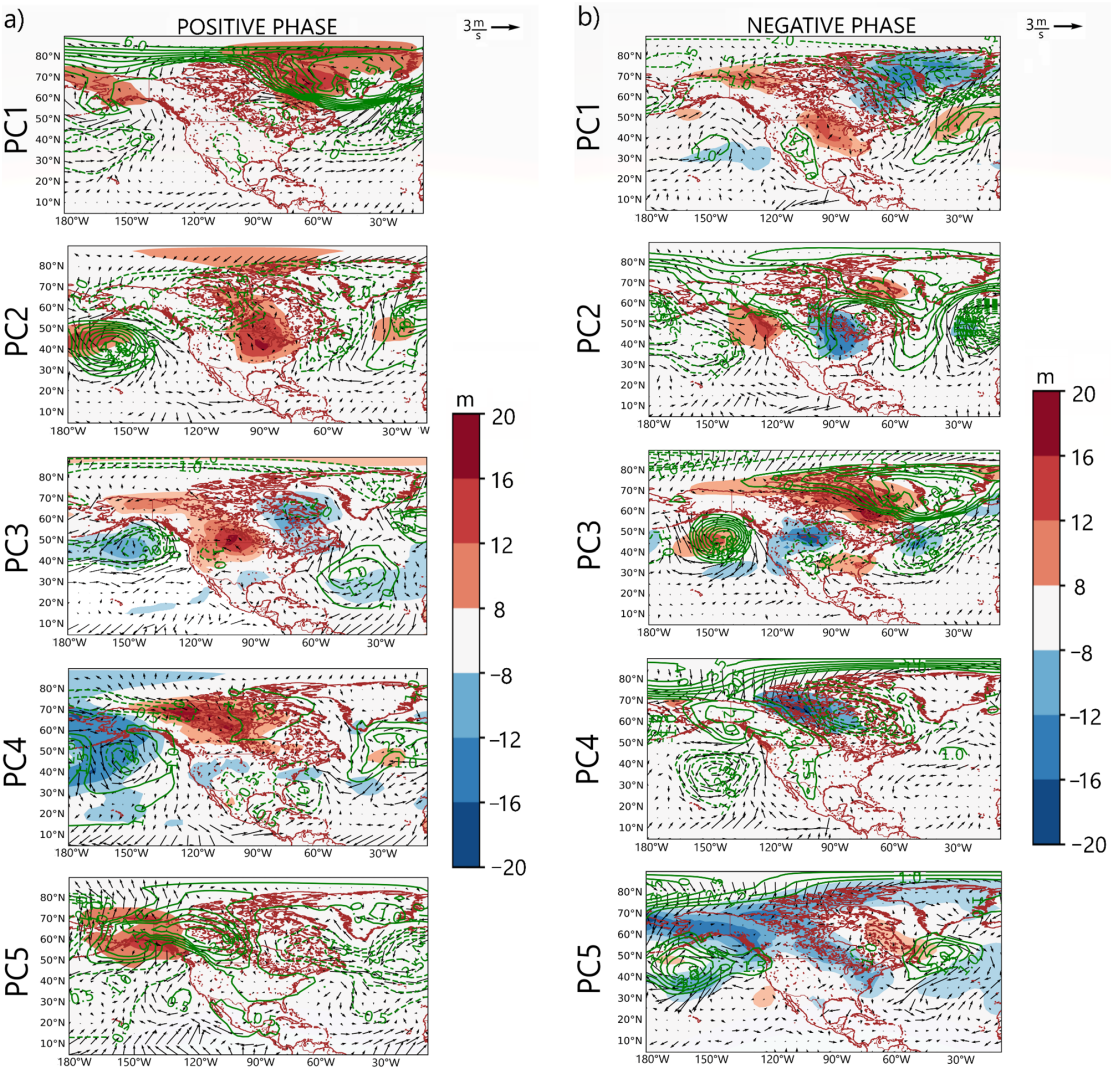
Composite anomaly maps of SLP (green contours), wind vector (black vectors), and atmospheric layer thickness between 1000 and 850 hPa (color) for the positive (**a**) and negative (**b**) phases of the temperature variability patterns in Fig. 1. Only values exceeding the 95-percentile confidence limit based on the permutation test are plotted Thick (dashed) contours indicate positive (negative) SLP anomalies.

**Figure 3:**
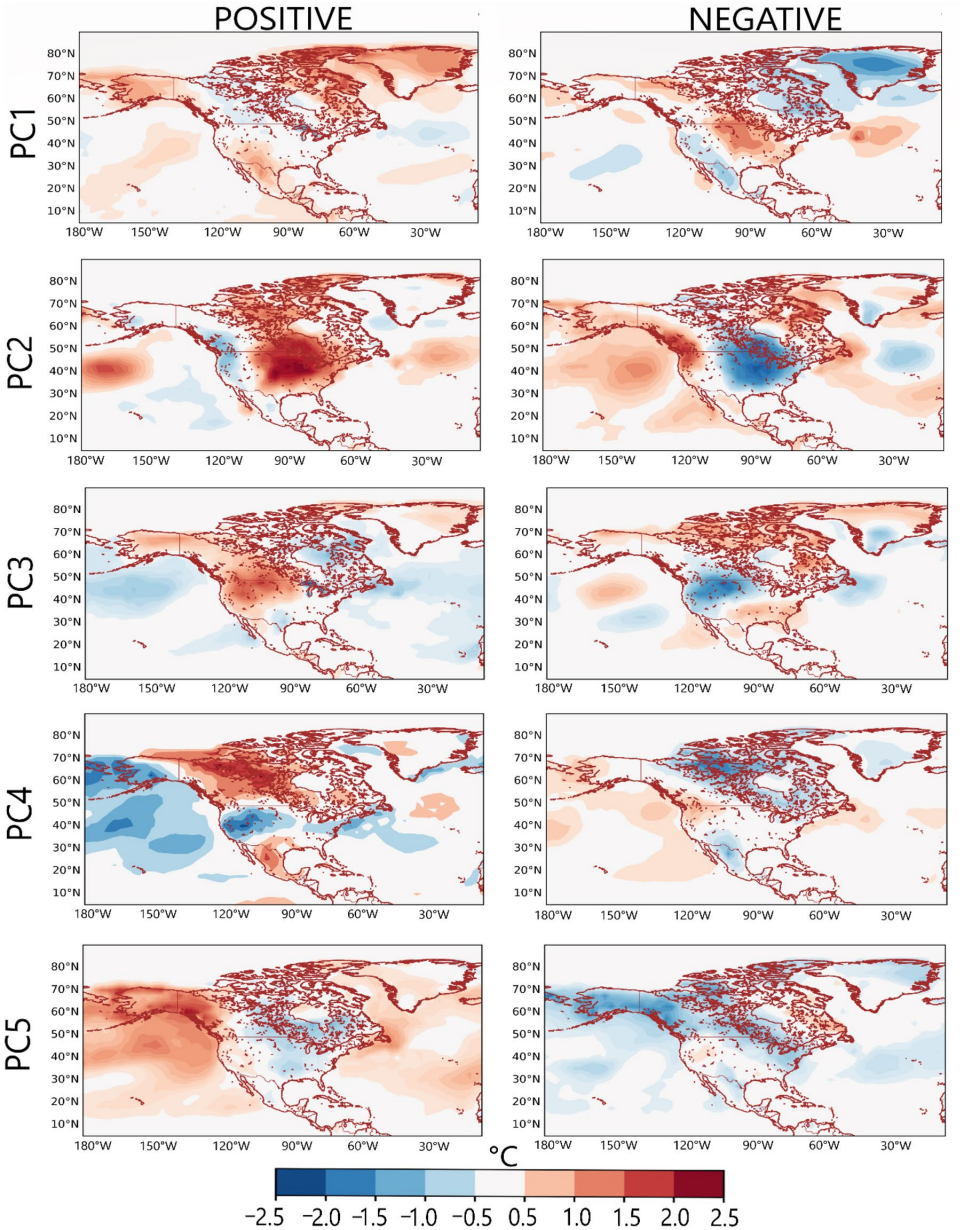
Composite anomaly maps of 2-m temperature over land and oceans, for the positive and negative phases of the temperature variability patterns in Fig. 1. Only values exceeding the 95-percentile confidence limit based on the permutation test are plotted.

From Fig. 1, overall, the temperature variability patterns from the three datasets are consistent. On average the patterns from CPC and GHCN are closest, with congruence matches mostly greater than 0.9. The patterns in Fig. 1 reveal relatively temperature-coherent regions during JJA. The loading magnitudes and isopleths reveal to what extent the grids are correlated in terms of JJA temperature anomaly. Regions with high loading magnitude are centers of action of the underlying circulation patterns associated with the variability pattern (Figs. 2 and 3). In our exploratory analysis, we structured the PC scores such that the positive phase designates the warm phase of the temperature variability pattern, and the negative phase designates the cold phase of the temperature variability pattern (Fig. 3).

PC1 is associated with temperature variability over Greenland and northeastern Canada, with weak loading magnitudes over Mexico and southern parts of the US (Fig. 1). From Fig. 2a, during the positive phase of PC1, an anticyclonic circulation anomaly dominates over Greenland while a cyclonic circulation anomaly can be seen over the Atlantic Ocean (south of Greenland), marking a meridional SLP anomaly pattern. The anticyclonic circulation anomaly over Greenland enhances warm air advection by southeasterly winds over Greenland, especially at the western parts (i.e., the positive thickness anomaly). Thus, the temperature anomaly is positive over Greenland (Fig. 3). Moreover, during summer, the anomaly climatological field of PC1 is often associated with high-pressure blocking over Alaska or western Canada; the absence of thunderstorms along with prolonged cloudless days, leading to heat waves over western Canada (Figs. 2 and 3). During the negative phase of PC1, a reverse circulation pattern can be seen (Fig. 2b) whereby cyclonic circulation anomaly dominates over Greenland and anticyclonic circulation anomaly dominates over the Atlantic Ocean, south of Greenland. The dominance of cyclonic circulation anomaly over Greenland enhances the penetration of Arctic air (i.e., the northwesterly winds) in Greenland (Fig. 2b), resulting in a decrease in atmospheric layer thickness and in below-average temperatures over Greenland (Fig. 3).

PC2 regionalizes the east-central parts of North America (Fig. 1). The negative loading seen in the western part (Fig. 1) suggests that the underlying circulation patterns associated with the development of the PC2 regime (Fig. 2, PC2) bring about an opposite temperature anomaly at parts of the western tips of North America (Fig. 3, PC2). From Fig. 2a, during the positive phase of PC2, a zonal SLP anomaly pattern is evident over the Atlantic Ocean—cyclonic circulation at the west coast of North America and anticyclonic circulation off the Atlantic coast. This increases warm air advection, thereby resulting in a positive temperature anomaly over the east-central parts of North America (Fig. 3, PC2). In the negative phase, the zonal SLP gradient implies cyclonic circulation anomaly in the Atlantic and anticyclonic circulation anomaly over the west coast of North America. The circulation pattern enhances the penetration of northwest Arctic air into east-central parts of North America resulting in decreased temperatures over the east-central parts of North America (Figs. 2b and 3, PC2).

PC3 regionalizes the west-central parts of North America (Fig. 1). During its positive (negative) phase, a cyclonic (anticyclonic) circulation anomaly can be seen off the northwest coast of North America, resulting in enhanced warm air (cold air) advection over the west-central parts of North America (Fig. 2, PC3); hence positive (negative) temperature anomaly is evident over the west-central parts of North America under PC3 (Fig. 3, PC3). However, it's important to note that, particularly during the summer, temperature variations in arid and semi-arid west-central North America may not be solely driven by these large-scale air advection patterns. The west-central parts of North America often experience high temperatures due to the increased solar radiation absorbed during long daylight hours and annual minima in precipitation. Additionally, the role of regional atmospheric dynamics, such as the comparatively mild southerly winds advecting moisture into the region, should also be considered. These winds often converge with a front of dry and warm air masses along the dryline, leading to the formation of thunderstorms. Thus, these factors, along with the circulation patterns indicated by PC3, contribute to the complex temperature variability observed in west-central North America.

PC4 regionalizes the North-central parts of North America (Fig. 1). From Fig. 2 during the positive (negative) phase of PC4, an anticyclonic (cyclonic) circulation anomaly can be seen in the northcentral part of North America, and cyclonic (anticyclonic) circulation anomaly over Alaska and the adjacent oceans. The circulation enhances warm air advection (cold air advection) by southerly winds (northerly winds) into the North-central parts of North America, resulting in positive (negative) temperature anomalies (Fig. 3, PC4).

PC5, from Fig. 1, regionalizes the north-western parts of North America (i.e., Alaska). The positive phase suggests a tripole SLP anomaly—over the northwestern land in North America, anticyclonic circulation is dominant, whereby over the oceans south and north of Alaska, SLP is weaker than normal, thereby inducing warm southerly winds over the north-western parts of North America (Fig. 2a, PC5) and resulting in above-average temperatures over the north-western parts of North America (Fig. 3, PC5). During the negative phase of PC5, anticyclonic circulation anomaly can be seen over the Beaufort Sea (North of Alaska), and the Gulf of Alaska (south); the resulting wind pattern over the north-western parts of North America is northerly winds, which enhance the penetration of cold Arctic air into the northwestern parts (Fig. 2b, PC5), thereby decreasing temperature over the north-western parts of North America (Fig. 3, PC5).

### Trends in the JJA temperature variability patterns

**Figure 4:**
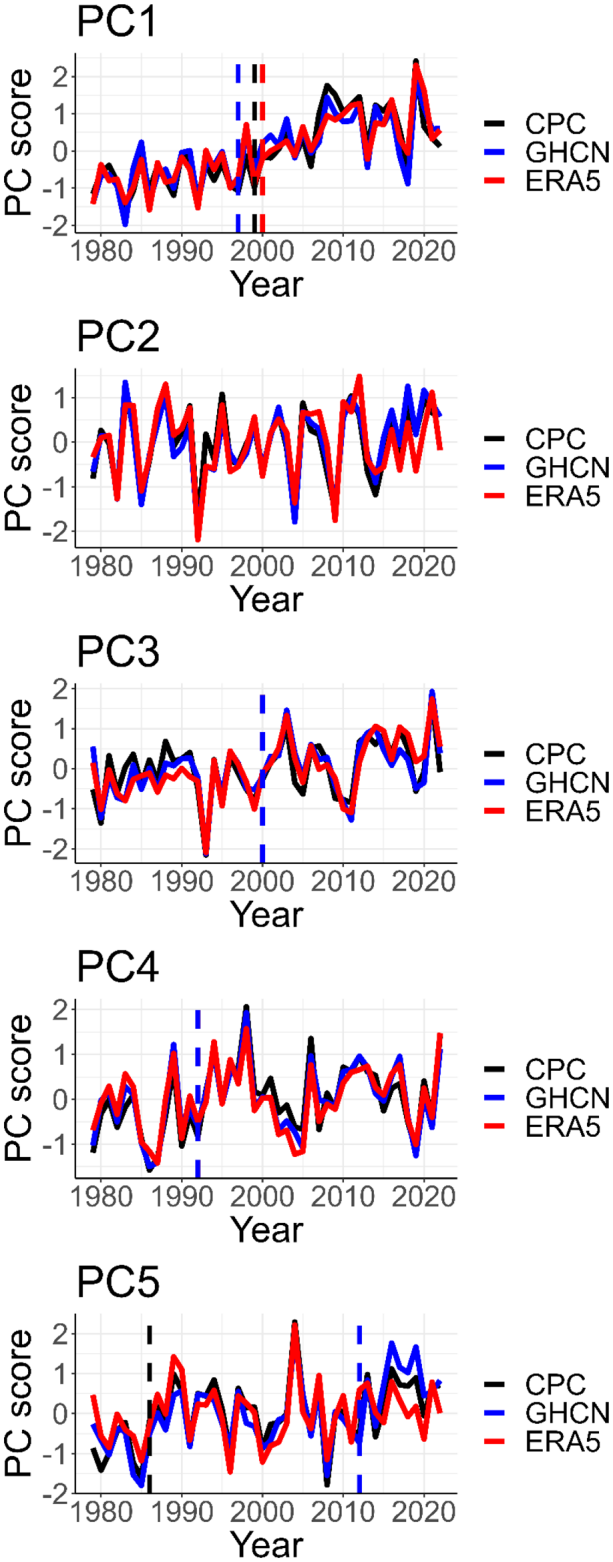
Time series of the JJA temperature variability patterns in Fig. 1 from 1979 to 2022. Time series is computed as the annual mean of the PC scores. A change point detection test that detects significant shifts in the mean of the time series was conducted with several methods in the trend package (R studio) [62]. Only statistically significant results at a 95% confidence level are reported (dashed vertical lines).

Figure 4 shows the time series of the temperature variability patterns in Fig. 1. To eliminate the subjectivity of the trends to data choice, the trends are considered statistically significant only when the modified Mann–Kendall test has a p < 0.05 from both CPC and GHCN. Using this criterion, the result from Table 2 shows that PC1, PC3, and PC5 have statistically significant positive trends in their amplitude—implying that their amplitudes are consistently signaling towards the warm regime—since the positive phases are structured to represent the warm regime (Figs. 2 and 3). Therefore, PC1, PC3, and PC5 are the variability patterns of interest that will further be analyzed in more detail. We recall from Fig. 1 that PC1 is associated with temperature variability over Greenland and northeastern Canada; PC3 is associated with temperature variability over the west-central parts of North America; and PC5 is associated with temperature variability over the northwestern parts of North America. Table 2 contains that in the long term, the amplitude of the circulations associated with the warm regime of these variability patterns has increased, and PC1 has the largest trend.

**Table 2:** Trends in the time series of the JJA temperature variability patterns in Fig. 1.

PC	CPC	GHCN	ERA5
PC1	+ 0.54*	+ 0.53*	+ 0.61*
PC2	+ 0.08	+ 0.23*	+ 0.02
PC3	+ 0.21*	+ 0.28*	+ 0.40*
PC4	+ 0.21*	+ 0.15	+ 0.13
PC5	+ 0.29*	+ 0.36*	+ 0.25*

The value in the Table is the Tau statistic from the modified Mann–Kendall test. Asterisks (*) indicate time series that are statistically significant (p < 0.05) and ± indicates the trend direction. Significant values consistent from GHCN and CPC are given in bold.

Furthermore, the time decomposition of the PC scores of the 3 PCs using moving averages resulted in the trend component and the oscillation/detrended component of PC1, PC3, and PC5. Tables 3 and 4 contain the correlation between the detrended components and the trend components of the 3 PCs and climate indices associated with North American weather patterns. The selected climate indices have significant correlations with the PCs at a 95% confidence level. From Table 3, the trend component of the three PCs is strongly correlated with the increase in global mean land and ocean temperature (GLMOT) with Kendall tau between ~ 0.5 and 0.8. For the three PCs, the trend components are also significantly correlated with climate indices designating boreal summer SST increase over the tropical Pacific Ocean—i.e., the positive phase of the Pacific warm pool region (PWPR) and the negative phase of the western Pacific index (WPI). The trend component of PC1 is also correlated with the positive phases of the Western Hemisphere warm pool (WHWP) and the Atlantic Multidecadal Oscillation (AMO). The trend component of PC3 is equally further correlated with the positive phase of the AMO, the negative phase of the North Pacific Pattern, and the negative phase of the Eastern Atlantic/Western Russia. Further examination of the PC scores using the spectral density method showed that the time series of PCs 1, 3, and 5 exhibit both interdecadal and interannual variability. The interdecadal variability is relatively highest under PC1 which might be linked to its association with the AMO (Table 4).

**Table 3:** Kendall correlation between the detrended component of the PC scores of PCs 1,3 and 5 and climate indices associated with North American weather patterns.

Index	PC1	PC3	PC5
AO	− 0.41	− 0.27	–
WP	–	− 0.23	–
SOI	–	–	− 0.30

Only statistically significant correlations at a 95% confidence level are reported. “–” indicates that the correlation is not significant. A detailed description of the climate indices in Tables 3 and 4 is available at https://psl.noaa.gov/data/climateindices/list/

*WP* Western Pacific, *AMO* Atlantic Multidecadal Oscillation, *EA/WR* Eastern Atlantic/Western Russia, *PWPR* Pacific warm pool area average, *GLMOT* global mean land/ocean temperature, *NPP* North Pacific pattern, *WHWP* Western hemisphere warm pool, *SOI* southern oscillation index.

**Table 4:** Kendall correlation between the trend component of the PC scores of PCs 1, 3 and 5 and climate indices associated with North American weather patterns.

Index	PC1	PC3	PC5
AMO	0.43	0.41	–
EA/WR	–	− 0.32	–
GLMOT	0.64	0.76	0.47
NPP	–	− 0.24	–
PWPR	0.48	0.57	0.37
WHWP	0.49	–	–
WP	− 0.33	− 0.40	− 0.28

Only statistically significant correlations at a 95% confidence level are reported. “–” indicates that the correlation is not significant.

**Figure 5:**
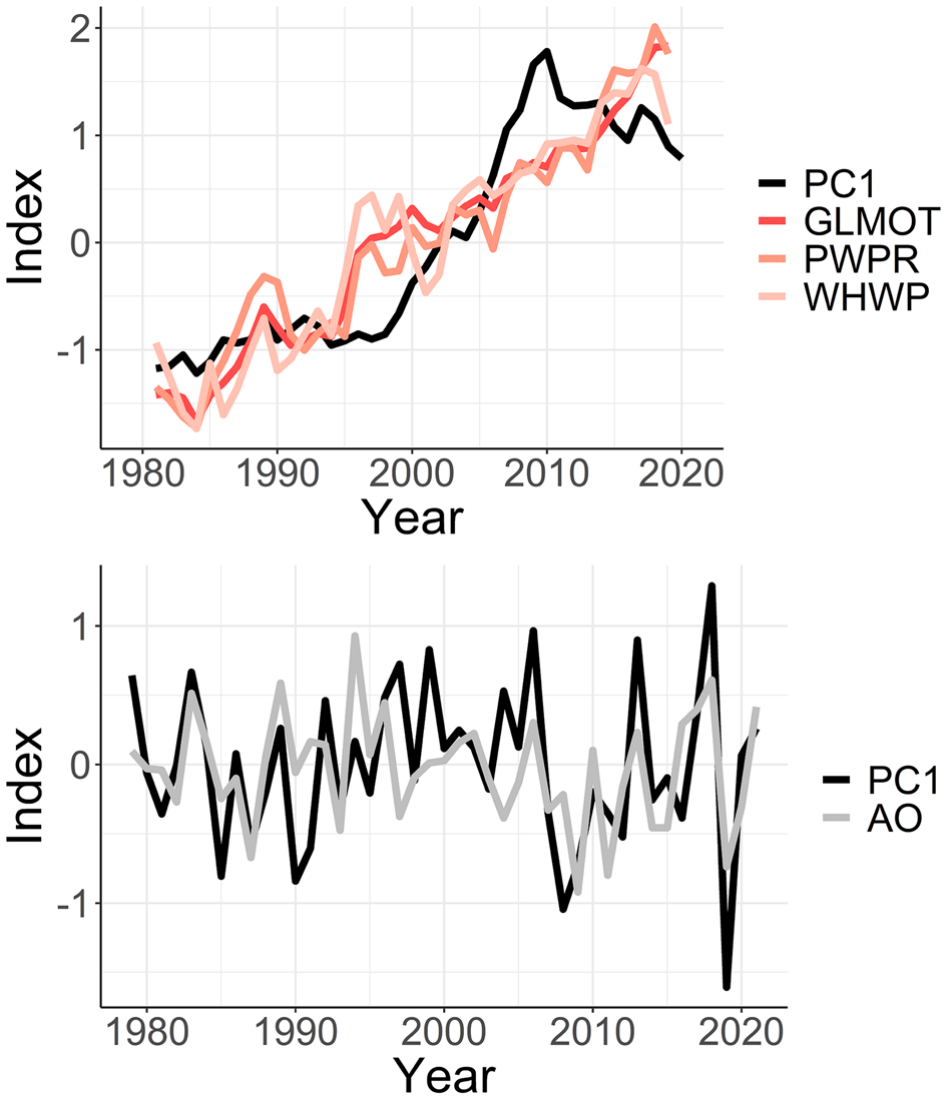
Five-year moving average of PC1 time series and time series of climate indices correlated with PC1, with Kendall correlation coefficient at least ~ 0.5 (top panel); and the detrended component of the PC1 and the AO index (bottom panel). PC1 was multiplied by minus 1 to keep it in phase with the AO index.

Partial correlation analysis showed that when the signal of the indices associated with warming of the tropical Pacific Ocean is controlled, the correlation between the PCs and GLMOT decreased (Table A1), which indicates why PC3 (that is more related to tropical SST anomalies) has the highest correlation with GLMOT. That is, the coupling between temperature variability in the regions associated with PCs 1, 2, 3 and GLMOT is strongly enhanced by the anomalous SST increase over the tropical Pacific Ocean. Further, the detrended component of PCs 1 and 3 is correlated with the Arctic Oscillation (AO) —Kendall Tau is ~ − 0.4 and − 0.3, respectively (Table 3). This indicates that the warming of Greenland and the west-central parts of North America at the inter-annual time scale is linked to the negative phase of the AO. In the short-term, temperature increase over the regions under PC3 are also associated with the negative phase of the WPI. Short-term temperature increase over the Alaska/northwestern region (PC5) is associated with the negative phase of the SOI (Table 3). As exemplified using PC1 that has the largest trend (Table 2), Fig. 5 (top panel) shows that over time, among other factors forcing the circulations, the long-term increase in the amplitude of the circulations associated with warm regimes is associated with changes in climate indices that the trend components are significantly correlated with. On the other hand, inter-annual variability in the amplitude of the circulation regimes is coupled with the climate indices that the detrended components are significantly correlated with (Fig. 5, bottom panel). The correlation between the (undecomposed) time series of the temperature variability patterns in Fig. 1 and climate indices are contained in Table A3. Also, Fig. A1 shows that the indices associated with PC1 in Tables 3 and 4, can be of predictive skill for its long-term changes. The predictions were less accurate for the inter-annual variability, suggesting that besides the large-scale circulations presented in this study, there are other (internal or regional processes) modulating the temperature variability patterns.

Further, PC1 and PC5 are negatively associated with the sea ice extent (Table A2), with Pearson correlation, R = − 0.83 and R = − 0.31, respectively, indicating that the decreasing sea ice over the Arctic is linked to enhanced anticyclonic circulations over the northern hemisphere high-latitudes, which increases temperatures over the high-latitude oceans (cf. Figs. 2 and 3, Positive phases of PC1 and PC5).

## Discussion

Arctic amplification has received significant attention regarding temperature changes in the northern hemisphere, owing to its disproportionate warming compared to other regions^31–35^. In line with existing literature, we found that during summer in North America, the amplitude of the temperature variability pattern associated with the warm regime over Greenland and the northeastern part of Canada (i.e., PC1) has the largest positive trend (Table 2 and Fig. 5, top panel). Several factors have been highlighted leading to the accelerated warming over Greenland. These include a reduction in the albedo due to the melting of sea ice^36–38^, the release of heat from the ocean^39^, and changes in atmospheric circulation patterns, such as changes in poleward energy transport^32^. We found that the warm regime over Greenland is associated with an anticyclonic circulation anomaly over Greenland and a cyclonic circulation anomaly over the Atlantic Ocean south of Greenland (Fig. 2, PC1). The anticyclonic circulation anomaly over Greenland increases warm air advection over Greenland; and the amplitude of the anticyclonic circulation has strengthened over time, with a significant increase in the mean state of the circulation at some time around 2000 (Fig. 4). Our result is in line with^40,41^: the positive temperature trends over Greenland are linked to anticyclonic circulations over the Arctic and Greenland. Also, the decrease in sea ice extent is strongly coupled with the positive phase of PC1 (R = $\left|0.83\right|$) —which contributes to 68% of the variance in sea ice extent during our analysis period. Therefore, the implying increasing anticyclonic circulation in the Northern Hemisphere high latitudes (associated with PC1 and PC5) are linked to the decrease in sea ice extent over the Arctic. Moreover, when sea ice melts, it exposes more of the ocean's dark surface, which absorbs more insolation and heats up. This additional heat can increase the temperature of the lower atmosphere, potentially influencing atmospheric circulation patterns through the ice-albedo feedback loop. Reference^42^ elucidated that an intensified anticyclonic circulation over Greenland and the Arctic Ocean, in conjunction with a barotropic structure in the troposphere, has the potential to boost downwelling longwave radiation above the ice. This is achieved by warming and adding moisture to the lower troposphere. We also identified a positive relationship between the long-term increase in anticyclonic circulation over Greenland and the positive phase of the AMO (Table 4). Thus, the general warming of the North Atlantic Ocean during positive AMO might be contributing to the long-term increase in anticyclonic circulations over Greenland.

A study by^43^ noted that accounting for the complex interplay between atmospheric circulations, Arctic Sea ice, and land ice changes, and internal climate processes is pivotal in refining climate models, particularly in the context of Arctic amplification, and ultimately improving the reliability of future projections under increasing greenhouse gas emissions. Therefore, the identification of the key circulation patterns associated with enhanced warming of the Arctic region and sea ice lost during boreal summer, in our study, can help improve the climate model simulations of the circulation patterns associated with Arctic amplification, and resultantly enhance the accuracy of climate model projections under future climate change.

Further^33^, reported that the Arctic amplification may be related to mid‐latitude weather; and several other studies have documented the link between the warming of Greenland and SST anomalies over the tropics^44–51^. Reference^49^ concluded that the slow-down of warming over Greenland (as can be seen in Fig. 5 towards the end of the analysis period) can be associated with the frequent occurrence of central Pacific El Niño events. Reference^50^ noted that wave-train patterns from the tropical Pacific propagate towards Greenland impacting temperature. Reference^50^ reported that the accelerated Arctic sea ice melt between 2007 and 2012 was linked to tropical Pacific SST changes. Reference^50^ detailed on the significant role of the Pacific-Arctic teleconnection, driven by SST changes in the tropical Pacific, on influencing Arctic atmospheric circulation and climate patterns. From 1979 to the early 2000s, warming trends were observed across the tropical SST, except over the tropical east-central Pacific, strengthening this Pacific-Arctic teleconnection and leading to increased Arctic warming and sea ice melting. This connection propagated through a Rossby wave train, is identified as a prominent internal mode linking the Arctic to lower latitudes. Consequently, it's largely associated with periods of expedited Arctic warming and sea ice loss, such as from 2007 to 2012. Herein, by decomposing the time series of the temperature variability pattern associated with Greenland, we found that the long-term warming over Greenland is significantly correlated with the warming of the tropical Pacific (Fig. A2 and Table 3), though the correlation is higher with GLMOT which may indicate that anthropogenic climate change is the more dominant driver (Table 3). The short-term temperature increase over Greenland, during JJA, is dominated by the negative phase of the AO. Due to the several climatic modes associated with temperature variability over Greenland, which can have distinct modulating effects, Fig. 5 shows that long-term changes in the amplitude of circulation over Greenland are non-linear. Finally, other regions with notable increases in the amplitude of the circulations associated with warm regimes are Alaska (PC5)^51^ and the west-central parts of North America (PC3). In those regions, the increase in global land and ocean temperatures is the dominant signal associated with the warming trend, in addition to the warming of the (western) tropical Pacific (Fig. A2).

## Conclusion

We have examined trends in boreal summer temperature variability patterns over North America from 1979 to 2022, as well as the climate indices contributing to the trends. Our major findings are:

- 1. Warm regimes over summer temperature coherent regions are generally associated with warm advection from lower latitudes, while cold regimes are associated with transport of the cold air from the higher latitudes.
- 2. The amplitude of circulation patterns associated with warm regimes has a positive trend over Greenland and Arctic Canada; the northwestern part of North America (Alaska); and the west-central parts of North America.
- 3. Anticyclonic circulations over Greenland and Arctic Canada and the northwestern part of North America (Alaska) are associated with above-average temperature over the regions; while cyclonic circulation over the Pacific coast, west of North America, play a role in temperature increase over the west-central parts of North America.
- 4. Increase in global mean land and ocean temperatures and SST warming over the tropical Pacific oceans are the major climate signals related to positive trends in the amplitude of circulations associated with warm regimes over Greenland and Arctic Canada; the northwestern part of North America (Alaska); and the west-central parts of North America
- 5. The rise in the strength and frequency of anticyclonic circulations over the Arctic contributes to approximately 68% of the variance observed in the diminishing extent of sea ice. This highlights the significant role that atmospheric patterns play in the ongoing reduction of Arctic sea ice coverage and enhances the potential of improving the accuracy of model simulation of the circulation patterns associated with Arctic amplification

In future research, it is anticipated that climate models can be utilized to conduct a detailed investigation into the impact of anthropogenic climate change on changes in these temperature variability patterns, considering different scenario forcings. The use of climate models may enhance the scientific community’s understanding of the underlying mechanisms driving these changes and their potential future trajectories.

## Data and methodology

### Data

To obtain gridded observational 2-m temperature data, we utilized three datasets: the Global Unified Temperature dataset from the Climate Prediction Center (CPC), available at https://psl.noaa.gov/data/gridded/data.cpc.globaltemp.html, the Global Historical Climatological Network (GHCN)^52^, and the ERA5 reanalysis^53^. Analysis using the three respective data sets helps characterize the sensitivity of the results to the choice of the analyzed data. This sensitivity arises due to the different methods used in creating the data. The observational data sets are more representative of the actual climate as they are based on station data^54^. However, following the interpolation to regular grids, using statistical models, the two observational data sets exist at a relatively lower horizontal resolution of 0.5° longitude and latitude compared to ERA5 reanalysis which has a horizontal resolution of 0.25° longitude and latitude. ERA5 is a product of data assimilation, and while it may not be as directly representative of specific weather events as observational data, it provides a consistent representation of global climate variables. Previous studies that evaluated ERA5 against station data in North America reported that it compares with other reanalysis data and equally performs well in capturing the statistics of station climate data^54^.

To investigate large-scale circulations associated with temperature variability patterns, we acquired sea level pressure (SLP) and 850 hPa wind vectors; and 850 hPa and 1000 hPa geopotential height datasets from the ERA5 reanalysis. SLP was chosen for this analysis because the gradients are proportional to changes in low-level horizontal advection. To characterize warm and cold air advection, the atmospheric layer thickness associated with 2-m temperature was calculated as the difference between the 1000 and 850 hPa height. All data sets were obtained from 1979 to 2022 at a monthly temporal resolution, except for the CPC dataset, which was originally obtained at a daily resolution before we computed monthly averages. The analysis period (i.e., from 1979) marks the post-satellite era with more reliable climate data sets as well as the common availability period of the data sets used in this study. We obtained climate indices that we related to the temperature variability patterns in North America from NOAA-PSL (https://psl.noaa.gov/data/climateindices/list/); only the climate indices that are significantly correlated with the PC scores at a 95% confidence level were selected for further analysis. The Sea Ice Extent data used in this study is obtained from the National Snow & Ice Data Center, an information and referral center that supports research in glaciology and related fields and manages and distributes scientific data. The specific dataset used is the Sea Ice Index, Version 3^55^.

### Methodology

**Figure 6:**
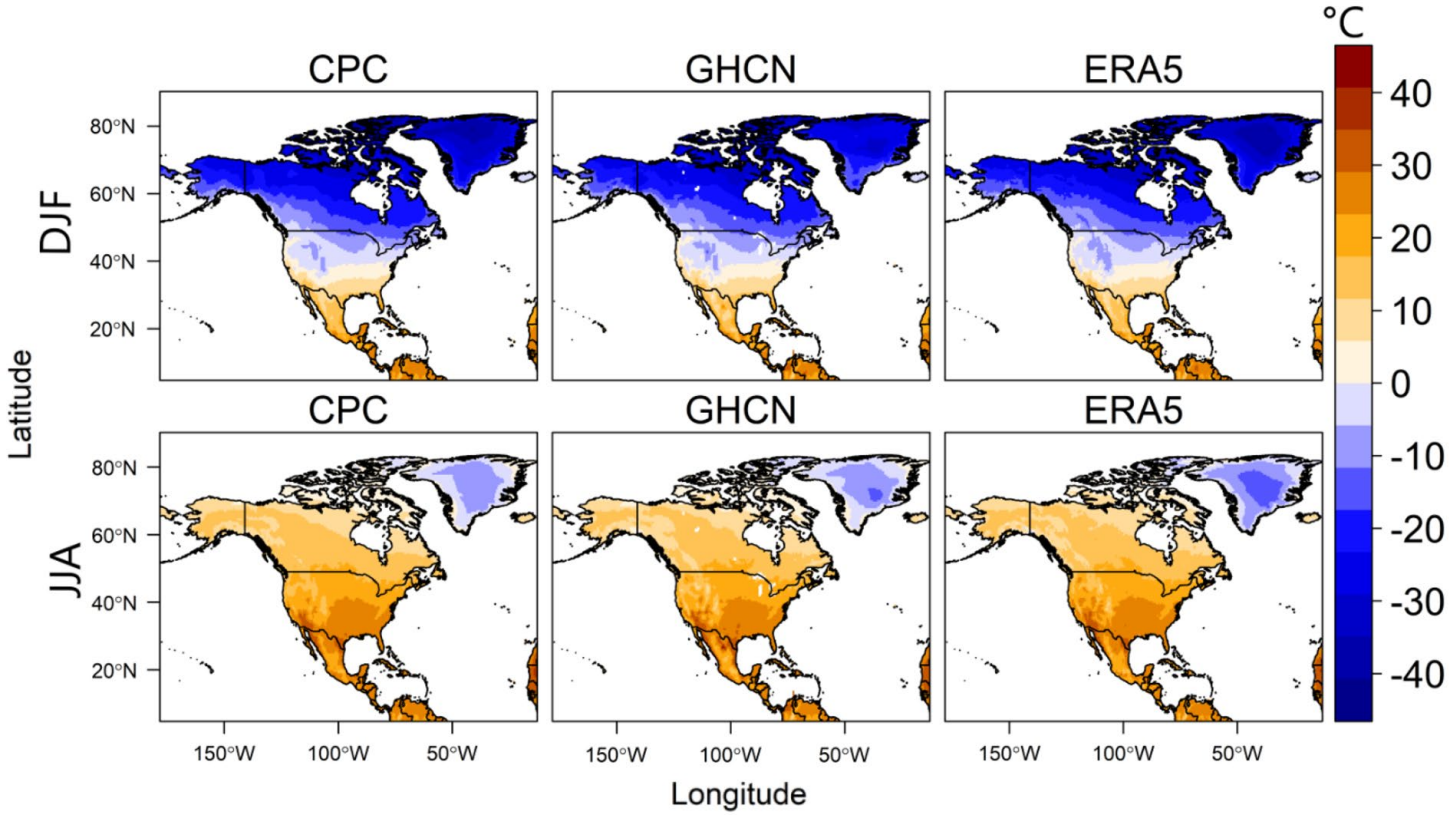
Seasonal climatology of temperature based on the 1992–2022 climatology.

Principal component analysis (PCA) is a widely used multivariate tool in climatology for removing noise in climate data and identifying (asymmetric) climatic modes of variability^56^. This is achieved through its variance maximization property and by post-processing the PCs with a suitable simple structure rotation algorithm^56^. In this study, we apply the rotated S-mode PCA^57^ to regionalize temperature in North America during boreal summer (JJA: July to August) and identify distinct temperature variability patterns. We focus on JJA because this season is associated with extreme temperature (warm) regimes in terms of magnitude and frequency (Fig. 6). Figure 6 also highlights the spatial variability of temperature in North America, emphasizing the need for a regional assessment of temperature variability and change in the region.

To ensure the consistency of the temperature variability patterns, the rotated S-mode PCA was independently applied to the three respective temperature datasets. In the pre-processing stage, the annual cycle was removed from the temperature datasets by subtracting the long-term mean of each month from the corresponding data values, for each grid point in Fig. 6. This step was taken to account for the annual variations in temperature due to seasonal changes, allowing for a more robust analysis of the interannual variability and long-term changes of the classified temperature variability patterns. Subsequently, rotated PCA was applied to the temperature anomaly data, with the trend component and inter-annual variability preserved.

Using the pre-processed temperature data over land in Fig. 6, the correlation matrix is utilized to obtain the relationship between the temperature field at the grid points in the study domain. Further, we applied the singular value decomposition to diagonalize the correlation matrix^56^, resulting in the PC scores, eigenvectors, and eigenvalues. The eigenvectors are spatial variability patterns, while the PC scores (time series) are the amplitude of the spatial variability patterns at a given time. The eigenvectors are multiplied by the square root of the corresponding eigenvalues to obtain the PC loadings, which are more responsive to simple structure rotation^56,57^. To enhance the physical interpretability of the PCs, we rotated the PCs using a set of oblique and orthogonal rotations to identify the most robust rotation algorithm that best maximizes the physical interpretability of the PCs. We applied the Varimax and Promax rotation algorithms, the former is an orthogonal rotation method that does not allow correlation between the PC scores while the latter is an oblique rotation method that allows correlation between the PC scores. We decided on the optimal number of PCs to retain and rotate. As noted by^56,57^, the arbiter for success in justifying the physical interpretability of rotated PCs is that they resemble the correlation matrix from which they are drawn—on the assumption that in physical sciences correlations can be physically interpreted or can be used to uncover physically meaningful relationships. Hence, we retained and rotated iteratively, 2 PCs and above; and using the congruence coefficient which is robust for matching Eigen-patterns^57,58^ the rotated PCs are matched to the correlation vector (i.e., from the correlation matrix) that indexes the highest loading magnitude on that PC. The solution that maximizes the physical interpretability of the PCs is that whereby all the retained and rotated PCs match the correlation vectors that they are indexed to, with a congruence coefficient of at least 0.92, which is the threshold that designates a good congruence match^56,57^. In the S-mode analysis, the correlation vector with the highest loading magnitude for a given PC designates the reference variability pattern in the correlation matrix that we seek to reproduce using the rotated PCs.

The decision regarding the number of PCs to rotate is based on the more reliable CPC and GHCN gridded observational datasets. The number of rotated PCs corresponds to the number of (relatively) JJA summer temperature coherent regions in North America, also referred to as the temperature variability patterns. As climatic regions do not have well-defined boundaries and may change gradually^59^, we have allowed for a fuzzy classification, meaning that a grid point may belong to more than one temperature region if the data permits. In a fuzzy classification, the isopleth indicates a gradual transition from one region to another, and some grids may not be classified if physically justifiable. It is important to note that the PC scores represent the amplitude of the temperature variability patterns. When the temporal pattern, represented by the PC score magnitude, oscillates to a high positive or negative value, notable temperature anomalies are expected over the corresponding temperature coherent region. To determine which phase of the PC score is associated with warm or cold conditions over the relative temperature coherent region, we calculate temperature anomalies relative to the 1992–2022 JJA climatology. We iteratively use distinct PC score thresholds to cluster dates (for the positive and negative phases of a PC, respectively) when the temperature regime is most pronounced. The optimal PC score threshold used to cluster representative dates is the one at which the composite anomaly pattern of the temperature data (i.e., the difference between the average temperature of the clustered dates and the JJA temperature climatology) matches the corresponding PC loading with a congruence match of at least 0.92. We also examined large-scale circulation anomaly fields associated with warm and cold regimes for each temperature variability pattern using composite anomaly maps of SLP, 850 hPa wind vectors, and atmospheric layer thickness between 1000 and 850 hPa. To assess the statistical significance of differences between the studied climate field and a long-term average, we employ the block permutation test. This method accounts for spatial and temporal autocorrelation, often present in climate data, which can infringe on the independence assumption of conventional statistical tests. By shuffling blocks of data, this approach preserves inherent data structures. This test generates a distribution of potential difference values under the null hypothesis (no difference), allowing for a comparative analysis with the observed difference to calculate the p-value. Statistical significance is then determined by a p-value less than 0.05.

The statistical significance of trends in the annual mean PC scores of all classified temperature variability patterns was assessed at a 95% confidence level using the modified Mann–Kendall test^60^. This test is non-parametric and accounts for autocorrelation in the data. Only temperature variability patterns with statistically significant trends, indicating consistent signaling towards the warm or cold regime, were considered for further analysis. To perform a robust analysis of the climate signal associated with long- and short-term variability in the amplitude of the circulation patterns (i.e., the PC scores), we performed time decomposition of the PC scores to obtain both the trend component and oscillations. We used moving averages, which is non-parametric. The optimal window size for the moving average is based on minimizing the root mean square error between the original data and the detrended data.

We used the Kendall correlation, which is a non-parametric and robust method, to assess the relationship between the trend component and climate indices available at https://psl.noaa.gov/data/climateindices/list/. This analysis aimed to identify the climate indices associated with long-term changes in temperature variability patterns. Similarly, we applied the same correlation between the climate indices and the detrended component of the PC scores to examine climate indices associated with the inter-annual or short-term variability of the PC scores. The statistical significance of the correlations was assessed at a 95% confidence level. To examine the predictive skill of the indices, for the PC scores, we applied the Long Short-Term Memory neural networks model. We divided the analysis period into two, we used one half to train the model, and the other for testing its predictions.

## Supplementary Information

Supplementary Information.

## Data Availability

The CPC and GHCN data sets are available at https://psl.noaa.gov/data/gridded. The ERA5 data set is available at https://climate.copernicus.eu/climate-reanalysis. The climate indices are available at https://psl.noaa.gov/data/climateindices/list/. The data sets can be requested by contacting Dr. Ibebuchi (cibebuch@kent.edu).

## Code availability

R studio and python were used for coding the methods as described in the methodology section.
