# Supplementary material for: Circulation patterns associated with trends in summer temperature variability patterns in North America
Source: Sci Rep. 2023 Aug 2;13:12536. doi: 10.1038/s41598-023-39497-5 (PMC10397208; doi:10.1038/s41598-023-39497-5)
Supplement: Supplementary file 1 — Supplementary Information. [file 41598_2023_39497_MOESM1_ESM.docx]

Table A1: Partial correlation between the raw PC scores of PC1 and climate indices. The values in parenthesis are original correlations between the raw PC scores and the climate index. Asterisk (*) marks correlations that are statistically significant at a 95% confidence level. Results are exemplified by PC1.

| Index | Controlled index | Partial Correlation [original correlation] |
| --- | --- | --- |
| AO | GLMOT | -0.35* [-0.34*] |
| PWPR | GLMOT | 0.17 [0.49*] |
| PWPR | AO | 0.51* [0.49*] |
| GLMOT | PWPR | 0.36* [0.60*] |

Table A2: Correlation between the raw PC scores of the JJA temperature variability patterns and climate indices. Only Correlations that are statistically at a 95% confidence level based on the Kendall Tau are reported.

| Index | PC1 | PC2 | PC3 | PC4 | PC5 |
| --- | --- | --- | --- | --- | --- |
| AMO | 0.35 |  | 0.34 |  |  |
| EA/WR |  |  | -0.32 |  |  |
| AO | -0.34 |  | -0.25 |  |  |
| GLMOT | 0.60 |  | 0.36 |  | 0.31 |
| NPP |  |  | -0.31 |  |  |
| PWPR | 0.41 |  | 0.35 |  | 0.33 |
| TNA |  |  |  | 0.39 |  |
| WHWP | 0.35 |  |  | 0.41 |  |
| WP | -0.33 |  | -0.34 |  | -0.31 |
| PNA |  | 0.37 |  |  |  |
| SOI |  |  |  |  | -0.21 |
| Sea ice extent | -0.83 |  |  |  | -0.31 |

*WP*: Western Pacific; *AMO:* Atlantic Multidecadal Oscillation; *EA/WR:* Eastern Atlantic/Western Russia; *PWPR*: Pacific Warm pool Area Average; *GLMOT:* Global Mean Land/Ocean Temperature; *NPP:* North Pacific Pattern; *Niño 1+2:* *SOI:* Southern Oscillation index; *WHWP:* Western Hemisphere Warm Pool; *TSA*: Tropical Southern Atlantic Index; *TNA*: Tropical Northern Atlantic Index


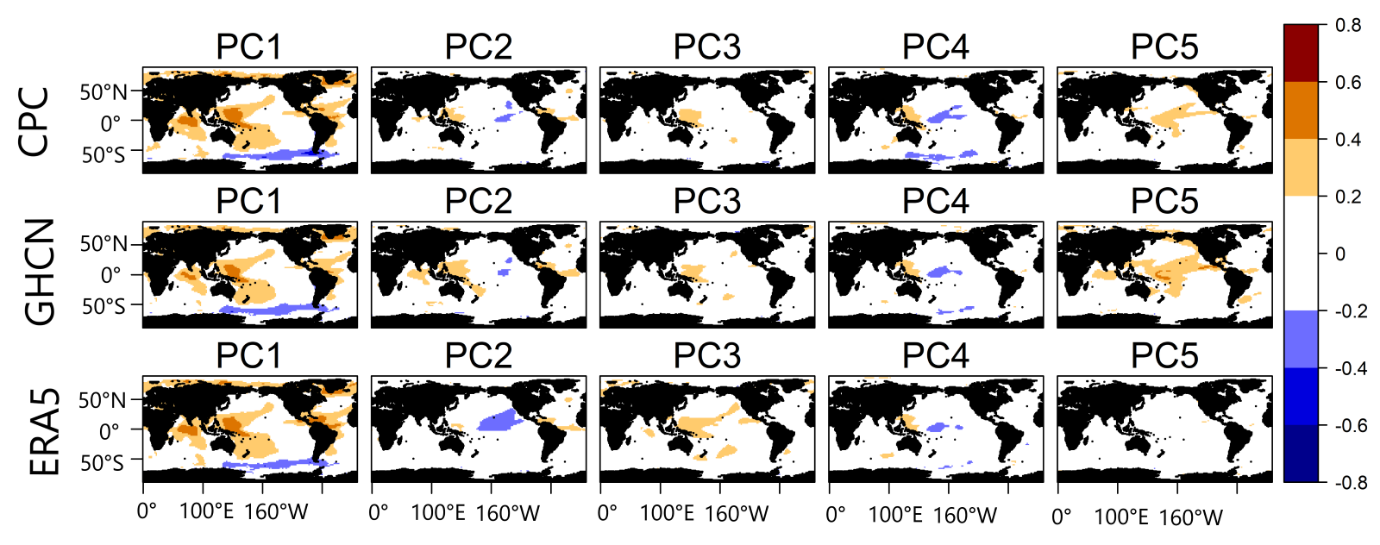


Fig. A1 Correlation between the temporal series (PC scores) of the JJA temperature variability patterns and sea surface temperature anomaly data during JJA.


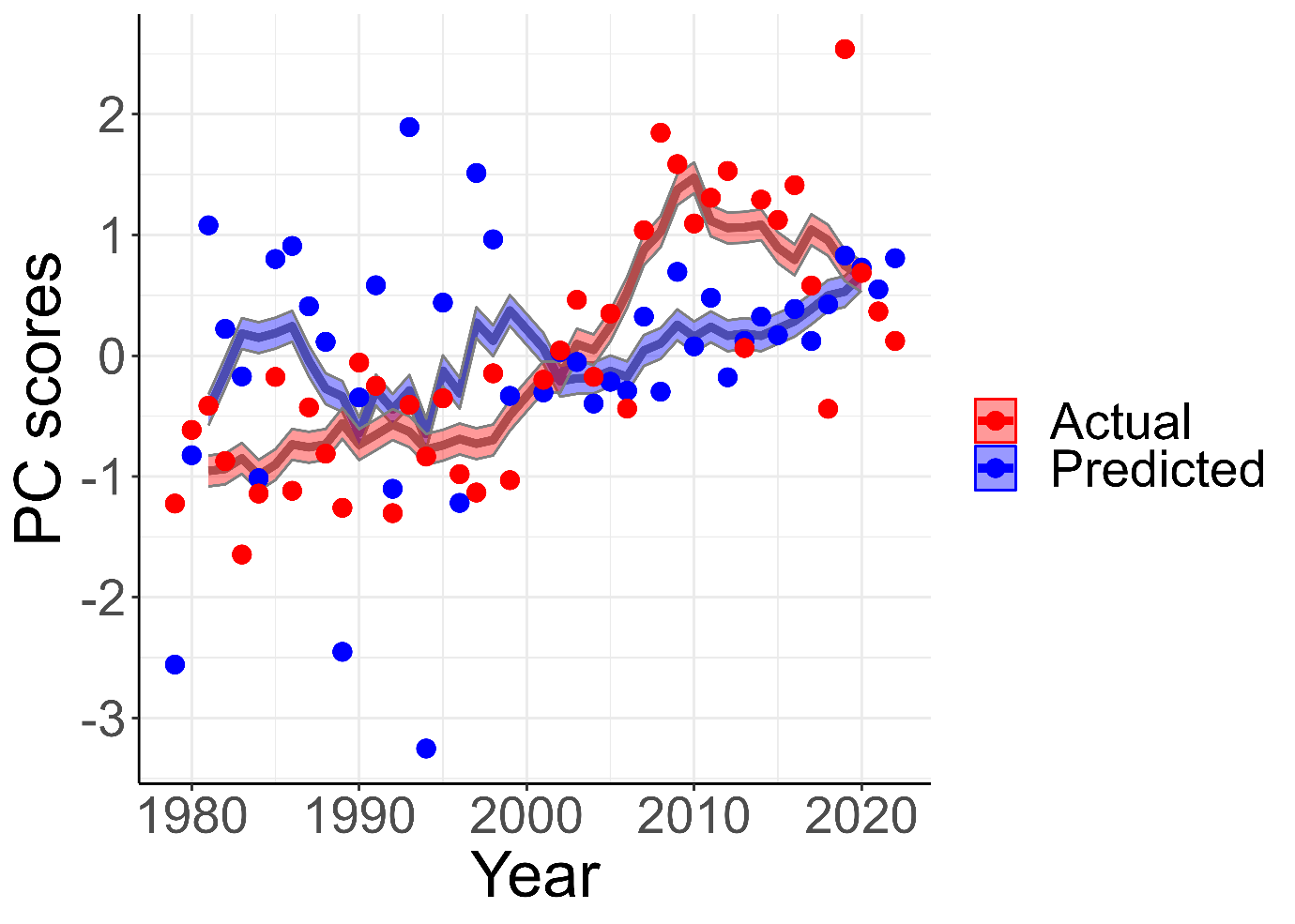


Fig. A2: Actual and predicted values of the PC scores of PC1 using the indices significantly correlated with PC1 in Tables 3 and 4. The model is trained for half of the analysis period and tested for predictions for the other half. Hence the PC scores of the entire analysis period was predicted.
